# 3D characterization of morphological changes in the intervertebral disc and endplate during aging: A propagation phase contrast synchrotron micro-tomography study

**DOI:** 10.1038/srep43094

**Published:** 2017-03-07

**Authors:** Yong Cao, Shenghui Liao, Hao Zeng, Shuangfei Ni, Francis Tintani, Yongqiang Hao, Lei Wang, Tianding Wu, Hongbin Lu, Chunyue Duan, Jianzhong Hu

**Affiliations:** 1 Department of Spine Surgery, Xiangya Hospital, Central South University, Changsha, 410008, China; 2 School of Information Science and Engineering, Central South University, Changsha, Changsha, 410008, China; 3 Pediatrics-Endocrinology. Johns Hopkins University, Baltimore, Maryland, 21203 USA; 4 Department of Orthopaedics, Shanghai Ninth People’s Hospital, Shanghai JiaoTong University School of Medicine, Shanghai, China; 5 Department of Orthopaedics and Traumatology, Nanfang Hospital, Southern Medical University, Guangzhou, 51000, P.R. China; 6 Department of Sports Medicine, Research Centre of Sports Medicine, Xiangya Hospital, Central South University, Changsha, 410008, China

## Abstract

A better understanding of functional changes in the intervertebral disc (IVD) and interaction with endplate is essential to elucidate the pathogenesis of IVD degeneration disease (IDDD). To date, the simultaneous depiction of 3D micro-architectural changes of endplate with aging and interaction with IVD remains a technical challenge. We aim to characterize the 3D morphology changes of endplate and IVD during aging using PPCST. The lumbar vertebral level 4/5 IVDs harvested from 15-day-, 4- and 24-month-old mice were initially evaluated by PPCST with histological sections subsequently analyzed to confirm the imaging efficiency. Quantitative assessments of age-related trends after aging, including mean diameter, volume fraction and connectivity of the canals, and endplate porosity and thickness, reached a peak at 4 months and significantly decreased at 24 months. The IVD volume consistently exhibited same trend of variation with the endplate after aging. In this study, PPCST simultaneously provided comprehensive details of 3D morphological changes of the IVD and canal network in the endplate and the interaction after aging. The results suggest that PPCST has the potential to provide a new platform for attaining a deeper insight into the pathogenesis of IDDD, providing potential therapeutic targets.

Intervertebral disc degeneration disease (IDDD) is an age-related process that has been identified as a leading cause of lower back pain^1^,^2^. The prevalence of IDDD has been shown to be as high as 85%, and some degree of IDDD appears to be present in all members of the aging population^2^,^3^,^4^. Considering the high economic cost (due to both health care services and absence from the workplace) associated with IDDD^3^, the importance of understanding the pathogenesis of IDDD to help develop more effective treatments cannot be overstated.

A disc is a functional anatomical unit consisting of three distinct anatomical parts: the centrally located intervertebral disc (IVD), including the nucleus pulposus (NP); the fibrocartilaginous annulus fibrosus (AF) and the superior and inferior endplates (EP)^5^,^6^,^7^. During aging, the disc undergoes a series of pathological processes, including cellular senescence and biomechanical and microstructural changes^8^,^9^,^10^. The IVD is the largest avascular organ in the body^6^, and nutrient transport to the IVD relies on diffusion through the adjacent endplates^11^. Nutrients are mainly supplied to the IVD through nutrition canals in the endplate, which is porous cancellous bone with cavities filled with marrow and blood vessels. The narrowing of the nutrition canals and insufficient nutrition delivery to IVD cells during aging due to the ossification of the endplate has been suggested as an initiating factor in disc degeneration^12^,^13^. Studies show that an interaction occurs between the endplate and IVD during the degeneration process^14^. Although the initial broad morphological changes have been evaluated using various methodological approaches, none of these studies reveal enough detail about the specific alterations in the endplate and IVD and their interaction during degeneration^15^,^16^. Additionally, the results obtained with conventional approaches, such as histology, rely on tissue sections, which can vary in terms of tissue thickness and sectioning orientation. The endplate and IVD are, however, highly specialized organs that have unique three-dimensional (3D) structures. The canals in the endplate are even more complex; they are branched and connected in longitudinally and transversely oriented patterns^17^. A more reliable strategy to evaluate the changes in the IVD and endplate would be to employ a 3D imaging method for the simultaneous visualization of these structures. With regard to the ossified endplate, the 3D morphological changes could be detected by conventional micro-CT (C-μCT)^12^,^17^, whereas magnetic resonance imaging (MRI) has advantages in soft tissue visualization that make it appropriate for IVD detection^18^. The IVD consists of soft tissue, whereas the vertebral endplate is characterized by the presence of a hyaline cartilaginous layer at a young age that becomes calcified after aging. Thus, the disc is a mixed tissue structure that cannot be simultaneously visualized in 3D utilizing the conventional methods mentioned above. The complex spatial and temporal micro-structural changes in the endplate and IVD, as well as their interaction after aging, are, to date, poorly understood, in part due to the lack of imaging characterization. A better evaluation of the morphological changes during aging is therefore critical for providing important insights for understanding the pathogenesis of IDDD and improving the current therapies that are used to treat IDDD. Accordingly, there is a high demand for an imaging method that allows for the simultaneous detection of complex mixed structures of sclerotic EP and soft IVD at a high resolution. Propagation phase contrast synchrotron micro-tomography (PPCST) is a novel imaging technique that combines the advantages of high spatial resolution, and 3D imaging^19^. It greatly improves the phase contrast of both soft and hard biological tissues and is thus useful for mixed tissue visualization^20^. Recently, the 3D anatomy of mixed tissue structures, such as the bone-tendon junction, tooth and ligament, have been successfully reconstructed from PPCST at high resolution^21^,^22^. Micro lesions of the cartilage of the femoral head in the rabbit have been evaluated using this technique^23^. PPCST also provided valuable information and allowed the analysis of the different components in printed hybrid cartilage constructs for cartilage tissue engineering^24^. Rodriquez *et al*. studied the 3D microstructure changes of the endplate in humans after degeneration using C-μCT^14^. However, there has been no previous study conducted to simultaneously elucidate the 3D micro-architectural changes in the endplate and IVD and their interaction after aging using PPCST.

In our study, we employed PPCST to simultaneously detect the spatial and temporal changes in the 3D architecture of the endplate and IVD and evaluate the age-related changes in the interactions between these two structures. This analysis will provide further insight into our understanding of the pathogenesis of IDDD and thus provide potential therapeutic targets.

## Results

### Comparison of images obtained from C-μCT and PPCST

**Figure 1:**
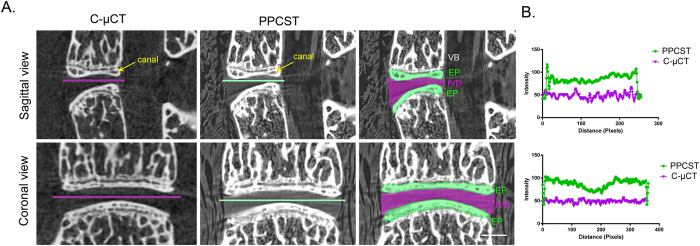
Corresponding sagittal and coronal sections acquired using conventional micro-CT (C-μCT) and propagation phase contrast synchrotron micro-tomography (PPCST). (**A**) The PPCST reconstructed images show a markedly superior soft tissue contrast of the intervertebral disc (purple) as well as the delineation of the endplate (green) compared to the C-μCT images. The canals in the endplate could be detected in both C-μCT and PPCST images. (**B**) A representative intensity profile across the dashed line drawing in the images in (**A**). Scale bar = 1 mm. VB = vertebral body, EP = endplate, GP = growth plate, IVD = intervertebral disc.

The 4^th^ and 5^th^ lumbar vertebrae obtained from 4-month-old mice were chosen to determine a suitable method for simultaneously visualizing the IVD and endplate. In the cross-sectional images produced by PPCST, the low-density soft tissue IVD between the 4^th^ and 5^th^ lumbar vertebrae was clearly visualized but was not detectable in the C-μCT images (Fig. 1A). The calcified endplate and the canals in the endplate were visible in the reconstructed images from both techniques. In addition, the distinct features of the surrounding soft tissues, e.g., the muscle and bone marrow in the vertebrae, were visible in the PPCST images (Fig. 1A). The intensity profile provided in Fig. 1B shows a sudden change in pixel intensity between the intramuscular spaces and the IVD from the PPCST images, while the density information of the line profile appears similar in the image obtained from C-μCT.

**Figure 2:**
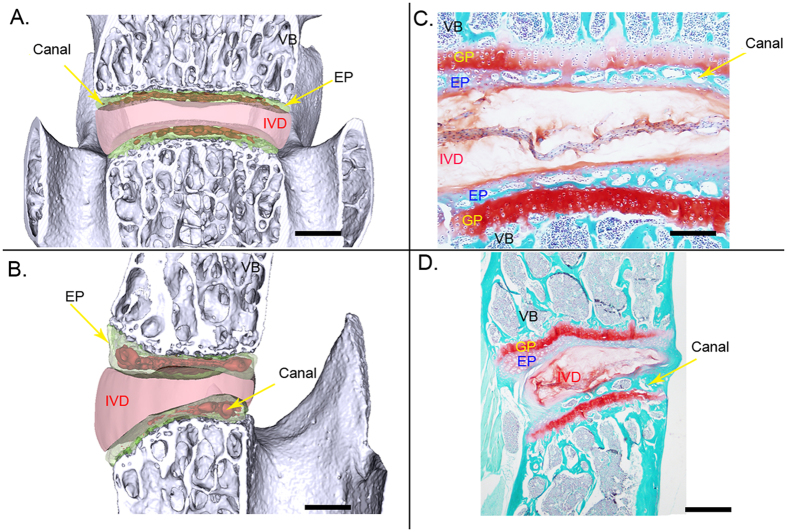
Comparison of the surface rendering images of the sagittal and coronal sections acquired with PPCST with images of histological samples. (**A** and **B**) Virtual sagittal and coronal sections acquired using PPCST. Canals located in the endplate and disc were clearly visualized. (**C** and **D**) Corresponding section images with Saf-O staining. Scale bar = 1 mm. VB = vertebral body, EP = endplate, GP = growth plate, IVD = intervertebral disc.

Virtual-cut coronal and sagittal images of the 4^th^ and 5^th^ lumbar vertebrae detected by PPCST are shown in Fig. 2A and B. The delicate structure and typical features of the canals in the endplate and the morphology of the IVD were simultaneously visualized owing to a strong refraction signal coming from the interface between the endplate and IVD. The features of the IVD and canals in the endplate obtained from PPCST were in agreement with the results acquired from the histological sections (Fig. 2C and D).

### Visualization of the 3D morphology of the canals in the endplate and IVD

**Figure 3:**
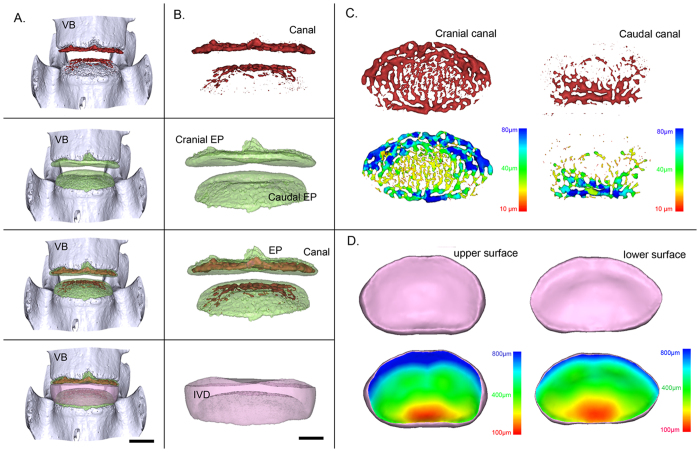
Representative 3D images of the canals in the endplate and IVD and corresponding pseudo-color images obtained by PPCST. (**A**) Intact 3D morphology of the lumbar functional unit. (**B**) 3D images of the canals, endplate, canals located in endplate and IVD. (**C**) Original and pseudo-colored images of the 3D canal network in the cranial and caudal endplates. (**D**) Original and pseudo-colored images of the upper and lower surfaces of the IVD. The pseudo-color bar in the lower-right corner indicates the diameter ranges of the canals in the endplate or the IVD thickness distribution.

Figure 3A and B show a representative 3D image of the 4^th^ and 5^th^ lumbar vertebrae, respectively, from a frontal view, with the segmented canal, endplate and IVD presented independently or in combination. Figure 3C shows a transverse view of the 3D canal network extracted from the cranial and caudal endplates. By using the technique described previously^25^,^26^, pseudo-color images were generated using a given color to represent the canal diameter. Under such conditions, it is possible to visualize changes in the canal diameter throughout the entire endplate. Additionally, the corresponding upper and lower surfaces of the 3D rendering of the IVD are shown in Fig. 3D. In the pseudo-color images, the colors represent the 3D thickness distribution of the IVD based on an analysis of the height variation across the entire IVD. The upper and lower surfaces of the IVD exhibited similar color arrangements, with slight differences that were characterized by more blue color in the anterior region of the upper surface than in the lower surface.

### 3D virtual canal micro-endoscopy

**Figure 4:**
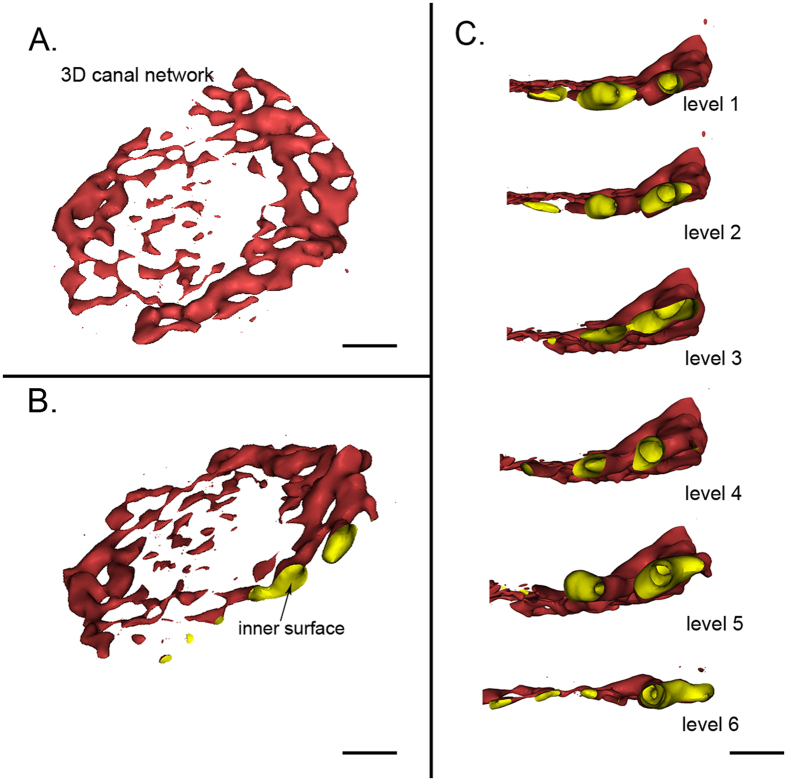
Virtual micro-endoscopy of the canals in the endplate. (**A**) Lateral view of the 3D canal network extracted from the endplate. (**B**) The inner surface of the 3D canal network is clearly present. (**C**) Virtual navigation within the multiple levels of the internal surface of the canal. (**A**) and (**B**) scale bar = 1 mm, (**C**) scale bar = 500 μm.

3D virtual micro-endoscopy is generated by the post-processing of PPCST data, and it offers a novel perspective for the visualization of the intraluminal surfaces of the canals. Based on the computer rendering, the 3D inner structure of the canals could be clearly detected (Fig. 4A and B). The canals were irregular, and the walls were mostly smooth and could be enlarged (Fig. 4B). Furthermore, a virtual endoscopy could provide a continuous intraluminal stereoscopic view that one could navigate along the inner surfaces of the canals within multiple levels (Fig. 4C).

### 3D morphology changes in the canal network in the endplate after aging

**Figure 5:**
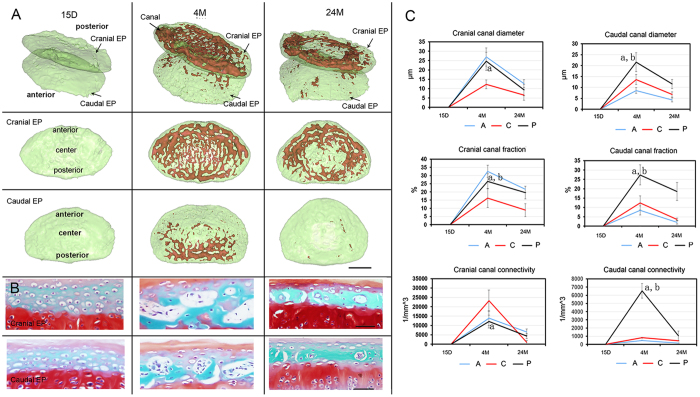
3D digitalized map quantitative information of the canal network for various ages. (**A**) Lateral view of 3D image of the canal network in the endplate after aging obtained using PPCST with the cranial and caudal endplates separately displayed. The endplate was divided into three different regions, anterior, center and posterior. (**B**) Representative image of the corresponding histology sections of the cranial and caudal endplates for various ages. (**C**) Quantitative analysis of the morphological parameters in the cranial and caudal endplates in three regions after aging. Scale bar = 1 mm; A = anterior; C = center; P = posterior. Data are the means ± S.D., n = 8 mice in each group. In panel (B), a indicates a significant difference between the posterior region and the center region, and b indicates a significant difference between the posterior region and the anterior region (p < 0.05, determined by two-way repeated measures ANOVA with Bonferroni’s post hoc test).

We next examined the 3D morphology changes in the canal network in the cranial and caudal endplates across different ages. As observed in Fig. 5A, the canals were not visible in the endplate in young 15-day-old mice, but they could be detected in 4-month-old mice. In comparison to the cranial endplate, no canals were observed in the anterior region of the caudal endplate. This finding is likely due to the difference in mechanical force bearing between these two endplates. Based on morphology parameter calculations, the canals in the cranial endplate have a larger diameter and fraction in the anterior and posterior regions compared to those in the center region (anterior vs center region, P = 0.0012; posterior vs center region, P = 0.0024). The distribution of the canal connectivity was, however, the reverse in the cranial endplate, with greater connectivity in the center compared to the anterior and posterior regions (center vs anterior region, P = 0.001; center vs posterior region, P = 0.004). In the caudal canal, a higher canal diameter, fraction and connectivity were found in the posterior regions in comparison to the center and anterior regions (posterior vs center region, P = 0.007; posterior vs anterior region, P = 0.0056). After aging, the canal in the center of the cranial endplate and caudal endplate disappeared, indicating that the endplate was undergoing calcification, which is consistent with the corresponding representative histological image (Fig. 5B). The morphological parameters of the canals measured in both the cranial and caudal endplates were significantly decreased in 24-month-old mice compared to 4-month-old mice (Fig. 5C).

**Figure 6:**
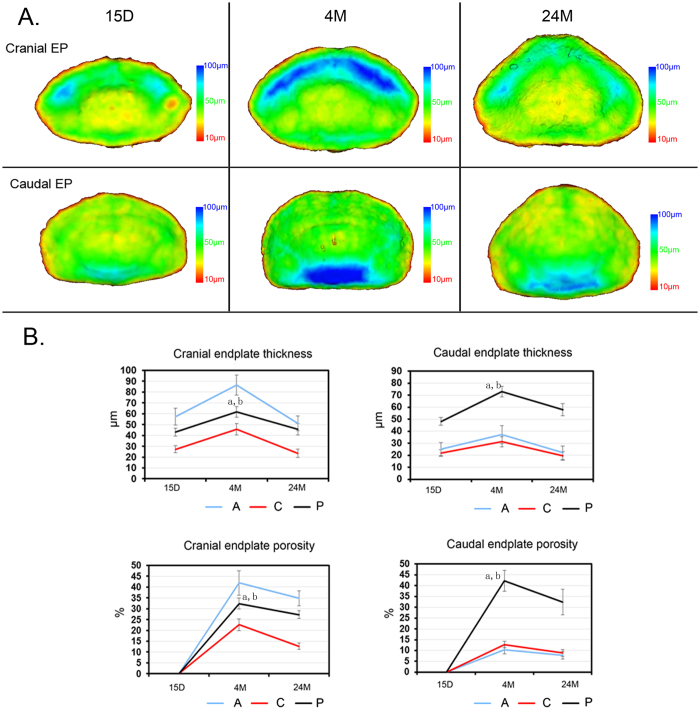
3D characterization of the endplate at different ages. (**A**) Vertical view of the 3D pseudo-color image of the endplate among mice of different ages. (**B**) Quantification of the endplate thickness and porosity after aging. A = anterior; C = center; P = posterior. The pseudo-color bar in the lower-right corner indicates the diameter ranges of the endplate thickness. Data are the means ± S.D., n = 8 mice in each group. In panel (B), a indicates a significant difference between the posterior region and the center region, and b indicates a significant difference between the posterior region and the anterior region (p < 0.05, determined by two-way repeated measures ANOVA with Bonferroni’s post hoc test).

To better visualize the changes in different regions in the endplate, pseudo-color images were generated. As observed, the endplate, labeled with yellow, was the thinnest centrally and opposite the anterior and posterior regions. In the endplates of 4-month-old mice, a blue color was observed at the anterior region of the cranial endplate, indicating that this region was the thickest. In contrast, the caudal endplate was thickest in the posterior region (Fig. 6A). The quantitative characterization of both the cranial and caudal endplate thicknesses was consistent with observations (Fig. 6B). An analysis of the endplate porosity demonstrated a similar distribution as that of the endplate thickness after aging (Fig. 6B). The results suggest that the thickness and porosity of the endplate are dependent on age, decreasing significantly during the aging process as an indicator that the shape of the endplate was condensed.

### 3D morphology changes in the IVD after aging

**Figure 7:**
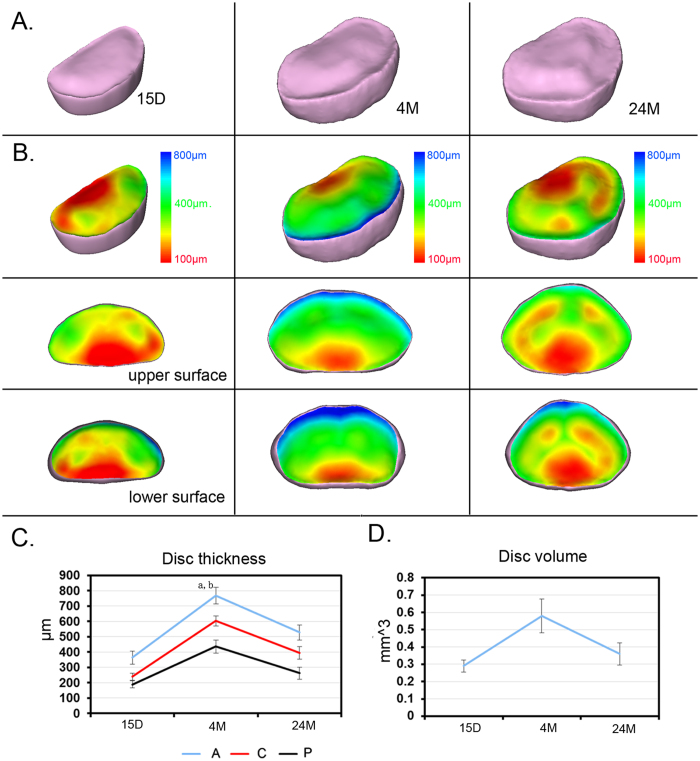
3D characterization of IVD at different ages. (**A**) Vertical view of the 3D images of the IVD from mice of different ages. (**B**) Corresponding pseudo-color image coding with the IVD thickness; the upper and lower surfaces of the IVD are separately displayed. The pseudo-color bar in the lower right corner indicates the diameter ranges of IVD thickness. The blue color represents the largest diameter, whereas the red indicates the smallest diameter. (**C**) Quantitative analysis of changes in volume and thickness distribution in anterior, center and posterior regions and of IVD for different ages. Scale bar = 1 mm, A = anterior; C = center; P = posterior. In panel (B), a indicates a significant difference between the anterior region and the posterior region, and b indicates a significant difference between the anterior region and the posterior region (p < 0.05, determined by two-way repeated measures ANOVA with Bonferroni’s post hoc test).

The 3D volume rendering image of the IVD across different ages and the corresponding pseudo-color image coding with the IVD thickness are displayed separately (Fig. 7A and B). As the figure shows, the morphology of the IVD differed across age groups, but the surfaces of both the upper and lower layers of the IVD exhibited the same gradient color changes, with blue in the posterior region and red in the anterior region (Fig. 7B). The IVD in mice of all ages was thinnest in the posterior region and thickest in the anterior region (Fig. 7B). In all regions of the IVD, including the anterior, center and posterior regions, the thickness initially increased, peaking at 4 months (4 months vs 15 days, anterior, P = 0.0008; center, P = 0.0001; posterior, P = 0.00015), and then decreased significantly after aging until 24 months (24 months vs 4 months, anterior, P = 0.00024; center, P = 0.0007; posterior, P = 0.00014) (Fig. 7C). A similar trend was found in the total IVD volume, indicating that the IVD space was narrowed during the aging process (Fig. 7D).

## Discussion

The pathogenesis of IVD degeneration is a complex process with a series of poorly understood molecular and biological determinants. The age-related degenerative changes in the endplate and IVD are closely related^27^,^28^ but have not been fully elucidated. This is the first study to simultaneously visualize in detail the alterations in the morphology of the IVD and endplate canal network during the aging process using PPCST. This technique successfully and distinctly captures the signs of IVD degeneration during aging and provides excellent 3D images of the IVD and canal network in the endplate. Furthermore, our findings provide comprehensive quantitative measurements of morphological parameters such as the canal diameter and connectivity in the endplate, as well as the IVD volume. Changes in these parameters with aging were associated with degeneration and are not easily detectable in conventional histological sections. In addition, unlike in conventional histological sections, this technique maintains the integrity of the specimen and can reveal the 3D morphology of the endplate and the IVD within the intact structure, which is more accurate and suitable for evaluating the pathogenesis of IDDD.

The nutritional support of the IVD remains central to the vitality of the IVD^29^,^30^. A number of studies have emphasized the importance of understanding the microstructure of the endplate, especially the canal network in the endplate^11^,^12^,^17^,^31^,^32^,^33^,^34^,^35^,^36^. It has been reported that nutrients are supplied to the IVD mainly via diffusion through the canals in the endplate^11^,^37^. Once the microstructure changes in the endplate, transport through the canal in the endplate is affected, impacting IVD metabolism^38^. In addition to the diffusion canals of the endplate, the vascularization of the endplate is also essential for the IVD nutrient supply^39^,^40^. Active blood flow in the canals within the endplate has been observed using vascular tracers^12^. Rui *et al*. described that osteoporosis could decrease the vascularization in the endplate, which subsequently exacerbates the degenerative process in IDDD^41^,^42^. Although PPCST is able to visualize the structure of the canals in the endplate, it does not differentiate the vasculature from other components, such as fat and bone marrow in the canal network. In the degenerated IVD, the endplate at the IVD-endplate interface appeared as solid bone in the C-μCT images, which could impede the nutrient supply to the IVD^12^. Although the importance of the nutritional supply to the IVD through the canals in the endplate has been recognized, there is limited information on the 3D morphological changes of the canal network during aging. In some animal models, the 3D microstructure of the canal network in the endplate has already been characterized by C-μCT^12^,^17^. The 3D microstructure of the canal network detected in our current study was consistent with previous findings in the sand rat^12^. The canal system in the endplate from the mouse was also a complex, interconnected 3D network. It was different from the Haversian canal system of cortical bone mentioned by Cooper^43^. However, additional studies are still required to uncover more valuable information about the morphological changes of the 3D canal network system in the vertebral endplate at various time points after aging. Some studies have reported that the disc degeneration after aging is associated with marked architectural changes in the endplate^12^,^44^; however, the complex relationship between the IVD degeneration and the endplate is not fully understood. Because the IVD and endplate constitute a mixed tissue structure, the simultaneous 3D visualization of the canal network and IVD during the aging process is a challenge using conventional methods. Our data provide experimental evidence that PPCST has great potential for mixed tissue visualization and can successfully detect the 3D morphology of the IVD and cartilaginous or calcified layer of the endplate from mice of different ages, a novelty that may provide valuable information regarding disc degeneration. We observed interactions between the 3D morphology changes in the intact IVD and canal network in the endplate. The characteristic morphological changes in the endplate from our study indicated that the endplate undergoes calcification and becomes dense during the narrowing of the canals after aging; in the meantime, the IVD becomes thinner. It is still not clear which mechanism underlies the observed changes in the IVD morphology and endplate structure or how the alteration of the endplate causes the degeneration of the IVD. The change in the IVD likely affects the biomechanical properties of the spine, causing aberrant loading to the endplate and leading to the calcification of the endplate^45^. However, the biomechanical alterations involved in this process are unclear. In our study, we found that the changes in the canal network disappear significantly in the center region of the endplate, indicating that biomechanical properties may play a role during this process. Changes in the endplate appear to occur early, which could block the diffusion of nutrients into the disc and subsequently trigger the onset of IVD degeneration. Nevertheless, additional studies are still needed to define the interconnectivity between the IVD and endplate during the aging process.

The imaging theory of the PPCST for examining mixed structures in the present study to produce the image contrast was based on the X-ray refractive properties after X-ray transmission from the specimen^46^. To achieve the desired image contrast between the soft IVD and the higher intensity of the refraction signal of the calcified endplate, the distance between the sample and the detector should be reasonable, as reported in a previous study^46^,^47^. Only by doing this can the absorption and phase contrast information of the subject be simultaneously visualized. This is the advantage of the PPCST technique when both types of tissue are of interest for imaging analysis. In addition to the advanced visualization properties, the contrast information of the structure can be extracted from the PPCST image and used for quantitative tissue assessments^48^,^49^,^50^.

The findings from the PPCST images provide essential information on the 3D microstructure of the canal network in the endplate and the 3D morphological changes in the IVD after aging, which establishes a new platform that may help gain insight into the pathogenesis of IVD degeneration. Although feasible, this technique inevitably possesses some limitations. We realize that aged mouse models are not the same as degenerated human discs, as upright bipedal walking is often associated with aberrant mechanical loading. It will be extremely interesting to conduct a preclinical study applying this technique to the 3D analysis of the microstructural changes of human discs during aging. However, the effective radiation dosed delivered by synchrotron radiation is higher than the conventional dose delivered by clinical CT scans, so the application of PPCST in clinical trials for humans is not yet possible. Work is ongoing to limit the radiation exposure in PPCST without compromising its finite imaging analysis, thus offering potential clinical applications.

In addition to the changes in the endplate, one of the main processes implicated in IVD degeneration is the diminished nutrition caused by changes in the subchondral bone under the endplate layer^42^. PPCST could potentially be applied to help uncover the 3D micro-structural changes in the subchondral bone and the interaction of this structure with the canals in the endplate or the IVD during the aging process. Additionally, a limited number of representative time points of ages of mice were selected to evaluate the feasibility of PPCST in the 3D characterization of the morphological changes in the intervertebral disc and endplate during aging. However, there is still a need for further exploration, with additional experiments providing a more systematic evaluation of the morphological changes in the IVD and endplate for more ages in mice using PPCST, which might help to predict the onset and progression of changes associated with disc degeneration and provide a more comprehensive conclusion.

In conclusion, our study revealed that PPCST is a promising imaging tool with great potential for providing comprehensive details about the 3D morphological changes in the IVD and the canal network in the endplate and their interaction during aging. The results suggest that the changes in the endplate occur early and may be associated with decreased nutritional support of the IVD at the onset of disc degeneration. The use of PPCST allowed us to directly characterize the 3D morphology of the IVD and the canal network in the endplate simultaneously and thus may provide detailed morphological information enabling a better understanding of the pathogenesis of IDDD with potential therapeutic implications.

## Materials and Methods

### Mice and Sample preparation

All animal experiments were approved by the Institutional Animal Care and Use Committee at Central South University (NO. 201503321, 03/01/2015) and were performed in accordance with relevant guidelines and regulations. C57BL/6 mice that were 15 days, 4 months or 24 months old (n = 8 for each) were purchased from Beijing HFK Bio-Technology (Peking, China) and housed at the Animal Centre of Central South University. All animals in each group were deeply anesthetized and euthanized with an overdose of 10% chloral hydrate intraperitoneally. The fourth and fifth level lumbar vertebrae (L4-L5) were harvested and fixed in 4% formalin and were subjected to C-μCT and PPCST analyses. After scanning, the samples were decalcified in 10% EDTA and then prepared for histological studies.

### Conventional μCT analysis

C-μCT scan images were captured at a spatial resolution of 7.4 μm on a Skyscan 1172 micro-computed tomography scanner (micro CT, Bruker, Kontich, Belgium) equipped with a 0.5-mm aluminum filter at a voltage of 55 kV, current of 180 μA, and power source of 10 W. The rotation step size used for acquiring images was 0.4°. NRecon and CTVol software were used for the transverse 2D cross-sectional reconstructions. Coronal and sagittal images were isolated in CTAn and loaded for 3D viewing in the Data viewer program supplied with the instrument. C-μCT demonstrated high spatial resolution for the endplate visualization, but poor soft tissue contrast for the IVD detection.

### PPCST scanning

After the C-μCT scanning, a sample at the same level of the IVD was then used for PPCST. The PPCST technique is based on Fresnel diffraction theory, which provides an edge-enhancement effect of the sample relative to C-μCT. When the detector is maintained at a proper distance from the sample, the inner structure of the sample can be visualized from the absorption of X-rays, and the phase shift induced by the intensity of the object can be recorded by the detector^51^.

The PPCST scanning of prepared samples was performed with the BL13W1 beam line at the Shanghai Synchrotron Radiation Facility (SSRF). X-rays were derived from an electron storage ring with an accelerated energy of 3.5 GeV and an average beam current of 180 mA. The size of the beam was approximately 45 mm (horizontal) × 5 mm (vertical), and a double-crystal monochromator with Si (111) and Si (311) crystals was used to monochromatize the X-rays.

The functional anatomical unit consisted of a mix of structures, including the soft IVD and calcified endplate. To enhance the contrast to show the difference in the density distribution between the two structures, the exposure time was set at 2.0 s, the scan energy was set at 15.0 keV, and the sample-to-detector distance was set at 30 cm after a series of confirmatory studies. After penetrating the sample, the X-rays were converted into visible light by a YAG:Ce scintillator (200 μm thickness) and digitized using a high-resolution 2,048 pixel × 2,048 pixel CCD camera with a physical pixel size of 7.4 μm (pco.2000, PCO AG, Kelheim, Germany). For each acquisition, 720 projection images were captured. Flat-field and dark-field images were also collected during each acquisition procedure to correct the electronic noise and variations in the X-ray source brightness. The projected images were reconstructed using a direct filtered backprojection algorithm performed by staff at the BL13W1 experimental station of the SSRF. Phase retrieval was applied and performed using phase-sensitive X-ray image processing and tomography reconstruction (PITRE) software^52^. After the phase contrast retrieval, a series of slice images were rendered into 3D images using VG Studio Max 3D reconstruction software and were quantitatively analyzed with the commercially available software (VG Studio Max (Version 2.1, Volume Graphics GmbH, Germany) and Image-Pro Analyzer 3D (Version 7.0, Media Cybernetics, Inc., USA)) to obtain quantitative data.

### Image analysis

**Figure 8:**
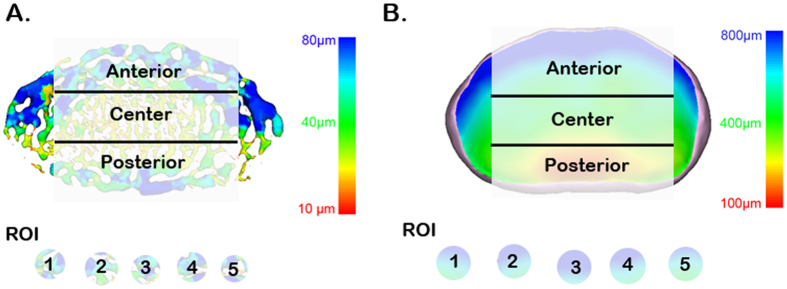
Representative PPCST image of a canal network in the endplate and IVD in a plane view. The vertical view was then further divided into anterior, central, and posterior areas. Five regions of interest were randomly selected to determine the average canal diameter, volume fraction and connectivity, and endplate porosity and thickness in each of these regions. The IVD thickness distribution was also calculated for these three regions. The pseudo-color bar in the lower-right corner indicates the diameter ranges of the canals in the endplate or the IVD thickness distribution.

To assess the morphological changes in the endplate and IVD after aging, the regions of interest (ROIs) in the endplate and IVD were selected in parallel in each age group of mice. The cranial and caudal endplate and IVD were analyzed separately. The vertical views of the endplate and IVD were divided into anterior, central, and posterior regions (Fig. 8). For each region, 5 randomly selected regions of interest (ROI) were drawn for analysis. The threshold algorithm available with Image-Pro Analyzer 3D was used for the ROI segmentation and the measurement of the structural morphometric parameters. The thickness distribution and volume of the IVD were determined. Because the endplate is present as a hyaline cartilaginous layer in young animals and becomes calcified during aging, the measurements were divided into two parts. The endplate thickness distribution was analyzed. The endplate porosity, diameter of the canal, canal volume over total volume of the endplate, and canal connectivity after aging were assessed.

### Histological observation

After the CT analysis, the vertebrae were re-fixed in 4% formalin, decalcified, dehydrated and paraffin-embedded. Sagittal and coronal sections were generated at a thickness of 5 μm, stained with Safranin O to delineate the endplate and IVD and photographed with an optical microscope (Olympus BX51, Tokyo, Japan). Histological images were then compared to reconstruction images produced by C-μCT and PPCST.

### Statistical analysis

Data are presented as the mean ± standard deviation (SD). Data analysis was performed with SPSS 17.0 (SPSS, Inc., Chicago, IL, USA). Significant differences among the different age groups were analyzed by two-way repeated measures ANOVA with Bonferroni’s post hoc test, and a *p*-value < 0.05 was considered to be significant.

## Additional Information

**How to cite this article:** Cao, Y. *et al*. 3D characterization of morphological changes in the intervertebral disc and endplate during aging: A propagation phase contrast synchrotron micro-tomography study. *Sci. Rep.* **7**, 43094; doi: 10.1038/srep43094 (2017).

**Publisher's note:** Springer Nature remains neutral with regard to jurisdictional claims in published maps and institutional affiliations.
